# Altered cerebral glucose metabolism normalized in a patient with a pediatric autoimmune neuropsychiatric disorder after streptococcal infection (PANDAS)-like condition following treatment with plasmapheresis: a case report

**DOI:** 10.1186/s12883-018-1063-y

**Published:** 2018-05-02

**Authors:** A. H. Nave, P. Harmel, R. Buchert, L. Harms

**Affiliations:** 1 Klinik und Hochschulambulanz für Neurologie, Charité-Universitätsmedizin Berlin, Berlin, Germany; 2 Berlin Institute of Health (BIH), Berlin, Germany; 3 Department of Diagnostic and Interventional Radiology and Nuclear Medicine, University Medical Centre Hamburg-Eppendorf, Hamburg, Germany

**Keywords:** PANDAS, PET-CT, Plasmapheresis

## Abstract

**Background:**

Pediatric autoimmune neuropsychiatric disorder after streptococcal infection (PANDAS) is a specific autoimmune response to group-A streptococcal infections in children and adolescents with a sudden onset of obsessive-compulsive disorders or tic-like symptoms. Cerebral metabolic changes of patients have not yet been observed.

**Case presentation:**

We present a case of an 18-year old male with a PANDAS-like condition after developing tic-like symptoms and involuntary movements three weeks after cardiac surgery. The patient had suffered from pharyngotonsillitis before the symptoms started. The anti-streptolysin O (ASO) titer was elevated (805 kU/l). Antibiotic therapy did not improve his condition. Intravenous immunoglobulins and high-dose cortisone therapy had minor beneficial effects on his involuntary movements. 18F-Fluorodeoxyglucose positron emission tomography/ computer tomography (18F-FDG PET/CT) demonstrated pronounced hypermetabolism of the basal ganglia and cortical hypometabolism. The patient was treated with five cycles of plasmapheresis. A marked clinical improvement was observed after four months. Cerebral metabolic alterations had completely normalized.

**Conclusions:**

This is the first report of cerebral metabolic changes observed on FDG-PET/CT in a patient with a PANDAS-like condition with a normalization following immunomodulatory treatment. Cerebral FDG-PET/CT might be a promising tool in the diagnosis of PANDAS.

## Background

Acute neuropsychiatric symptoms in children and adolescents can have multiple causes, including autoimmune reactions following a preceding microbial infection. [1] Pediatric Acute-onset Neuropsychiatric Syndromes (PANS) can be triggered by infection (pediatric infection-triggered autoimmune neuropsychiatric disorders, PITANDS) or have non-infectious metabolic, or environmental triggers. [2] PITANDS are frequently caused by group A beta-hemolytic streptococcal (GAS) infections [3], which has been coined pediatric autoimmune neuropsychiatric disorder after streptococcal infection (PANDAS) by Susan Swedo and colleagues in 1998. [4] In PANDAS, it is hypothesized that antibodies directed against streptococcal antigens cross-react with surface proteins of the basal ganglia activating calcium calmodulin-dependent protein kinase II (CaMKII), hence causing altered central dopamine neurotransmission. [5] Additionally, it is thought that specific strains of S. pyogenes causing a strong immune response must meet a genetic predisposition of infected children that lead to autoimmune reactions with cellular and humoral immune responses. [6] Most recently, findings of a large-scale study support the PANDAS hypothesis, demonstrating an increased risk of mental disorders, particular OCD (obsessive-compulsive disorders) and tic disorders, in young individuals with GAS throat infections. [7]

So far, published imaging findings of patients diagnosed with PANDAS are mainly restricted to magnetic resonance imaging (MRI) describing increased volumes of the basal ganglia. [8, 9] One study could demonstrate increased microglia-mediated neuroinflammation in the basal ganglia on positron emission tomography (PET) using a ^11^C-[R]-PK11195 tracer. [10] To our knowledge, no data exist on the use of fluorodeoxyglucose (FDG) PET in patients with PANDAS. Here, we report the first case with a PANDAS-like condition that received a FDG-PET/CT before and after treatment with plasmapheresis.

## Case presentation

A male, 18-year old patient presented at the Department of Neurology at the Charité – University Hospital Berlin, in February 2016 because of involuntary movements and neuropsychiatric symptoms.

Involuntary movements included orofacial dyskinesias and tic-like symptoms, dysarthric voice accompanied by dysphagia, and hyperkinetic movements of the extremities with jerking and dystonic components that were predominantly present on the left side of his body.

Six months earlier, in August 2015, the patient, who had a congenital bicuspid aortic valve with aortic distension, underwent surgical replacement of the aortic valve and the ascending aorta using a cardiopulmonary bypass system and mild hypothermia. The remaining medical history was unremarkable without pre-existing neuropsychiatric conditions.

Precisely 3 weeks after surgery, the patient experienced the acute onset of an emotional dysbalance, hyperactivity, and loss of concentration accompanied by involuntary movements of his left upper extremity, especially his left hand. Because of further deterioration of the involuntary movements, now extending to his left leg and causing gait instability; worsening of his mood state with increasing aggressiveness at home; sleeping problems with frequent nightmares; and a severe decline in school performance the patient was admitted to a clinic in November 2015. He was reported to have had symptoms of pharyngotonsillitis days before symptoms initially started. The anti-streptolysin O (ASO) titer was elevated at 805 kU/l (reference values: < 200 kU/l). Further laboratory tests including anti-basal ganglia antibodies, CSF analysis, and a cranial CT scan showed unremarkable results.

Assuming a post-streptococcal neuropsychiatric disorder, the patient was treated with high-dose penicillin (3 × 1 Mio. I.E./ d) for three days without any clinical effect. An immunomodulatory therapy with intravenous immunoglobulins (IVIG) with a dose of 2 g per kg of bodyweight (105 g in total) was applied showing a minor, short-lasting improvement of his involuntary movements. He was discharged home on a symptomatic, anti-dopaminergic therapy with tiapride 100 mg TID. Tiapride mildly improved his sleep quality, but induced dizziness during daytime.

On presentation at the Charité several weeks later, the patient showed further psychological deterioration revealing depressive moods, attention deficits, and progressive decline in school performance, threatening his graduation. He described having vivid nightmares and a loss of body weight (5 kg in 2 months, i.e. 9% of body weight). The ASO titer was still elevated at 450 kU/l, whereas anti-deoxyribonuclease B (Anti-DNaseB) titer, autoimmunological parameters, and CSF analyses remained unremarkable. In particular, cerebral autoantibodies (a large panel antibodies including anti-NMDA receptor- and anti-TPO-antibodies) could not be detected neither in serum nor in CSF. Immunohistologically, plasmapheresis eluate of the patient did not reveal any specific or unspecific binding on murine brain tissue. Cerebral magnetic resonance imaging (MRI) showed small bicerebellar and left frontal microbleeds, but no focal lesion or specific pattern of atrophy. Electroencephalography (EEG) displayed diffuse brain dysfunction without further implication.

**Fig. 1:**
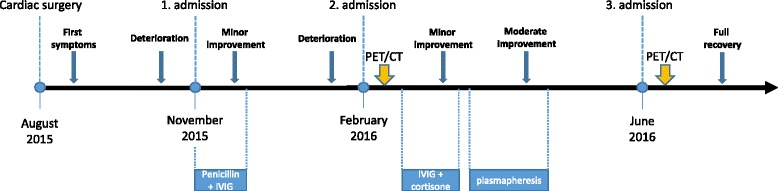
Timeline of the clinical course of the presented case. First symptoms started three weeks after cardiac surgery. Intravenous immunoglobulin and cortisone treatment resulted in only minor and transient improvement of symptoms. Clinical stabilization was first observed after plasmapheresis. Normalization of impaired cerebral glucose metabolism measured via PET/CT was achieved four months later

Comprehensive neuropsychological testing identified a dysexecutive syndrome characterized by a decrease in working memory capacity, attention, and concentration deficits, as well as frequent failure of spontaneous speech. Psychosomatic counseling revealed several underlying family-based conflicts. His mother was described as a controlling person who discounted his symptoms and persistently browsed through his personal belongings. He described himself as sad with fears of separation and being lonely. Suicidal thoughts had occurred two months prior to the second admission. The clinical course of the patient is depicted in Fig. 1.

The differential diagnoses at this time were: 1) Sydenham chorea minor (SC), 2) Pediatric Autoimmune Neuropsychiatric Syndrome after Streptococcal infection (PANDAS) or PANDAS-like condition, 3) antibody-mediated autoimmune encephalitis (e. g anti-NMDA receptor encephalitis) 4) Psychosomatic disorder, 5) Post pump chorea. [11]

**Fig. 2:**
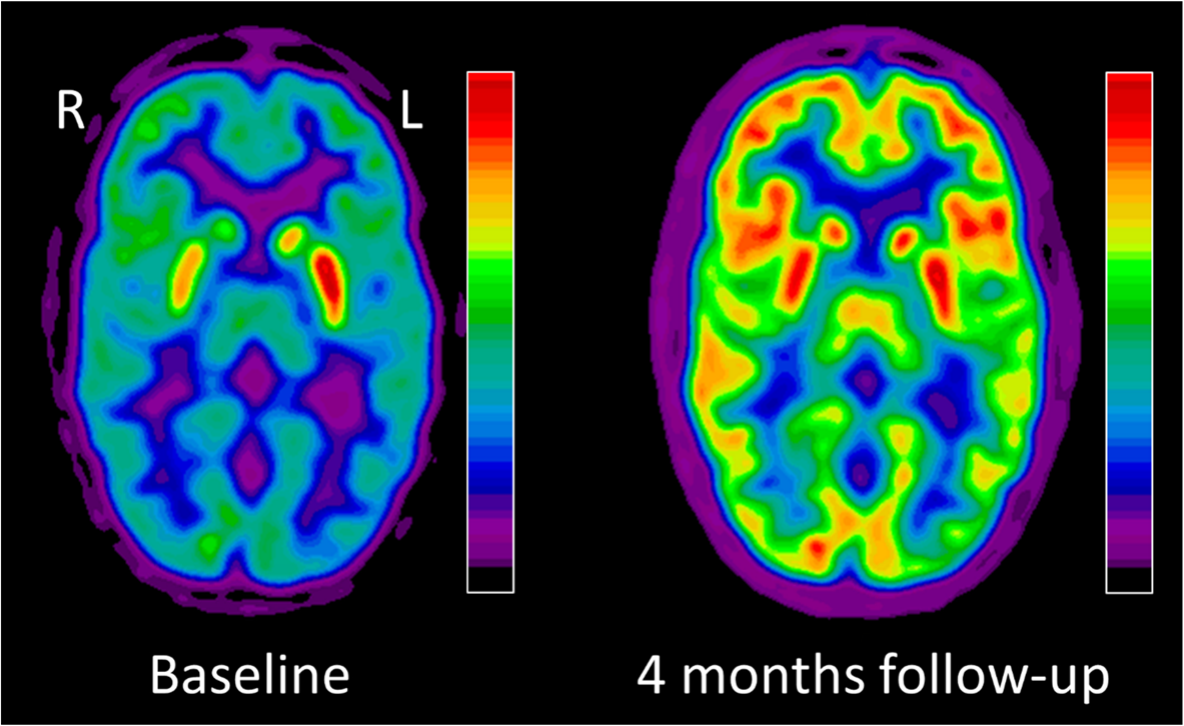
Images of the cerebral FDG positron emission tomography/ computer tomography (PET/CT). At baseline prior to plasmapheresis, the patient demonstrated a moderate to severe hypermetabolism of the left striatum. The cortex revealed hypometabolic signal. Metabolic changes were completely normalized at follow-up four months later

Diagnostic work-up was expanded and a cerebral FDG positron emission tomography/ computer tomography (PET/CT) demonstrated a moderate to severe hypermetabolism of the basal ganglia, especially of the left striatum, whereas the cortex revealed hypometabolic signals (Fig. 2).

The anti-dopaminergic medication was discontinued and an additional IVIG therapy had marginal effects on his symptoms. A series of high-dose cortisone therapy (1 g i.v.) for five days improved his restlessness, muscle strength of his left arm, and quality of sleep, but symptoms persisted. Subsequently, we initiated five cycles of plasmapheresis, and ASO titer significantly decreased (78 kU/l).

Four months later at follow-up, the patient demonstrated a normalized neurological exam with a minimal fine motor skill deficit in his left hand. Neuropsychological disorders had resolved. Follow-up FDG-PET/CT revealed a complete normalization of cerebral glucose metabolism (Fig. 2). The ASO titer remained at normal levels (197 kU/l). The patient’s personality returned to its premorbid state and family-based stress factors had dissolved. He resumed taking psychotherapeutic sessions twice a month.

## Discussion and conclusions

We report the case of an adolescent patient diagnosed with a PANDAS-like condition that showed severe striatal hypermetabolism and cortical hypometabolism on FDG PET/CT imaging. Consistent with clinical improvement, glucose metabolism completely normalized four months after immunomodulatory therapy with five cycles of plasmapheresis. To the best of our knowledge, this is the first report of a patient with a PANDAS-like condition demonstrating changes of glucose metabolism before and after treatment.

Opposed to surgical intervention [12], the beneficial effects of immunomodulatory therapies, such as IVIG and plasmapheresis, in OCD/tic disorder patients have been reported previously. [13–15] Although IVIG administration could not demonstrate statistically significant effects compared to placebo in a large clinical trial, the application was safe and well tolerated in all treated patients. [16] We also did not experience any complication during IVIG or plasmapheresis therapy. Despite the reported benefits, several clinical guidelines do not support the use of immunomodulatory therapies in patients with PANDAS limiting their clinical use. [17] However, others support its use in severe cases as a second-line therapy after inefficiency of antibiotic treatment. [18, 19]

We assume that a PANDAS-like condition was the most appropriate diagnosis for our patient. According to the published diagnostic criteria on PANDAS, patients must meet the following criteria: 1) abrupt onset of OCD/tic-like symptoms or severely restricted food intake, 2) prepubertal onset of symptoms, 3) acute symptom onset and episodic (relapsing-remitting) course, 4) temporal association between Group A streptococcal infection and symptom onset/exacerbations, and 5) association with neurological abnormalities. [20] In our case, the patient experienced an acute onset three weeks after cardiac surgery. Additional to orofacial dyskinesia and tic-like symptoms, he demonstrated neuropsychiatric symptoms including obsessional fears, separation anxiety, depressive mood, sleep and body weight problems as well as dramatic decline in school performance. Because of the age of the patient and the lack of a positive throat culture, a PANDAS diagnosis is not justified. Striatal hypermetabolism was described in SC [21, 22], however, post pump chorea, presenting in children following open-heart surgery, was shown to be associated with hypometabolism of the basal ganglia. [23] In addition, patients described with post pump chorea developed symptoms within the first two weeks after surgery and were much younger (age < 3 years). Other differential diagnoses such as atypical manifestations of an anti-NMDA receptor encephalitis or Hashimoto’s encephalitis must be mentioned. However, negative antibody titers in both blood and CSF as well as the absence of immunohistological findings on murine brain tissue, and the cerebral distribution of metabolic changes on FDG-PET/CT make these diagnoses unlikely. [24–26]

In conclusion, PANDAS is a severe disorder that needs appropriate treatment with immunomodulatory therapy, if antibiotic treatment is not effective and symptoms progress. FDG PET/CT seems to be a valuable diagnostic approach to prove cerebral metabolic alterations in PANDAS. Future cohort studies should assess the sensitivity of FDG PET/CT in diagnosed PANDAS patients and investigate the association of metabolic abnormalities with severity of clinical symptoms.

## Abbreviations

*ASO*: anti-streptolysin O
*FDG PET/CT*: FDG positron emission tomography/computer tomography
*GAS*: group A beta-hemolytic streptococcus
IVIG: intravenous immunoglobulins
OCD: obsessive-compulsive disorders
*PANDAS*: Pediatric Autoimmune Neuropsychiatric Disorder after Streptococcal Infection
SC: Sydenhams’s Chorea
